# Computing the Expected Cost of an Appointment Schedule for Statistically Identical Customers with Probabilistic Service Times

**DOI:** 10.1155/2014/949726

**Published:** 2014-01-30

**Authors:** Dennis C. Dietz

**Affiliations:** CenturyLink, Inc., Boulder, CO 80301, USA

## Abstract

A cogent method is presented for computing the expected cost of an appointment schedule where customers are statistically identical, the service time distribution has known mean and variance, and customer no-shows occur with time-dependent probability. The approach is computationally efficient and can be easily implemented to evaluate candidate schedules within a schedule optimization algorithm.

## 1. Introduction

Consider a medical service system where a fixed number of patients are to be scheduled for appointments with a single provider over a finite time horizon [1]. The common service time distribution for every patient is known, as is the time-dependent probability that any patient will fail to show up for the appointment. During the time each arriving patient waits for service, an institutional waiting cost of *c*_*w*_ per hour is incurred. Expected waiting times can be kept small by planning large gaps between appointments, but the gap sizes are constrained by a finite provider availability period. To ensure that all patients are served, the availability period can be extended at an institutional overtime cost of *c*_*o*_ per hour. The objective is to determine a schedule of patient arrival times that will minimize the expected total cost (waiting and overtime) of operating the appointment system.

Similar problems could arise in many other operational contexts. For example, a maritime shipping entity may need to optimally schedule vessel dockings within a fixed window of rented dock time (overtime penalties for docks can be quite severe). A surgical suite manager may need to determine a schedule for procedures that minimizes expected waiting times for surgical teams while avoiding intrusion on a subsequent high-priority commitment. Analogous to medical environments, providers in legal, financial, or other personal service professions that operate on an appointment basis are usually concerned with both server efficiency and client waiting times.

Appointment optimization has received substantial attention in operations research literature, originating with a brief reference by Herne in 1951 in the discussion following Kendall's important article on queueing theory [2]. Numerous attempts followed to obtain reasonable scheduling rules through simulation modeling, beginning with Bailey [3]; relevant surveys are presented in Magerlein and Martin [4], Przasnyski [5], and Ho et al. [6]. Analytic modeling efforts began with steady-state approximation of appointment systems, where statistically identical customers could be scheduled to arrive on a continuous time horizon [7–9]. Transient models and solution methods were later addressed [10–14]. The mathematical complexity of optimization in continuous time led other researchers to constrain arrivals to discrete time points (e.g., a 15-minute lattice) [15, 16]; operationally, this approach was more realistic for most applications. Service time distributions were assumed to be identical and follow exponential, Erlang, or Coxian probability distributions. Vanden Bosch and Dietz [17] generalized the service time model and proposed a heuristic sequencing algorithm for statistically nonidentical customers. Kaandorp and Koole [18] subsequently derived an exact sequencing and scheduling algorithm for customers having exponentially distributed service times with distinct mean values.

Consistent with the standard practice of scheduling appointments on a lattice of time slots with fixed width Δ, assume that each of *N* customers is appointed to enter the service queue at one of *K* discrete times 0, Δ, 2Δ,…, (*K* − 1)Δ. All customers arrive exactly on time, and the server works under a first-come-first-served (FCFS) discipline whenever one or more customers are in the system. Let *a*_*k*_ be the number of appointments scheduled at time (*k* − 1)Δ, and define a *schedule* as a vector *S* = (*a*_1_, *a*_2_,…, *a*_*K*_) such that ∑_*k*=1_^*K*^ *a*_*k*_ = *N*. Any single component of *S* has a feasible range of 0 to *N*. For example, a trivial problem where *c*_*w*_ = 0 and *c*_*o*_ > 0 clearly yields an optimal schedule of *S* = (*N*, 0,0,…, 0).

The first customer should always be scheduled in the first time slot, since a later arrival would waste server time with no improvement in waiting times. The total number of schedules that must be considered is the number of ways in which the remaining *N* − 1 appointments can be assigned to *K* time slots, which can be computed as (_*N*−1_^*N*+*K*−2^). Hence, a typical problem involving 20 customers and 60 time slots would generate (_19_^78^) = 6.71 × 10^17^ candidate schedules. In general, the large number of candidate schedules will prohibit an optimality search by exhaustive enumeration. Assuming that a method exists for determining the total cost *C*(*S*) of any schedule, optimality can be efficiently achieved using the algorithm below [1].

1. Determine a schedule that is assured to have a lower cost than all earlier schedules, *S*_*E*_, using the following procedure.
  - Establish an early incumbent schedule *S*_*E*_. If no better bound is available, let *S*_*E*_ = (*N*, 0,0,…, 0).
  - Let *m* be the largest integer for which the *m*th arriving customer in *S*_*E*_ is not scheduled at time (*K* − 1)Δ.
  - Establish a candidate early schedule *S* by shifting the arrival of the *m*th arriving customer in *S*_*E*_ by Δ later, unless this shift causes the order of customer arrivals to change. If all customers but the first are scheduled at (*K* − 1)Δ, stop (recall that the first customer's arrival is fixed at zero).
  - If *C*(*S*) < *C*(*S*_*E*_), let *S*_*E*_ = *S* and return to step 1(b).
  - If *m* > 2, decrement *m* and return to step 1(c). Otherwise, each customer of the current *S*_*E*_ but the first has shifted without improvement, and *S*_*E*_ is established.
2. Establish a candidate late schedule *S*_*L*_ by shifting each arrival time by Δ, if feasible. Perform a parallel procedure to step (1) (shifting arrivals by Δ earlier rather than later).
3. If *S*_*E*_ and *S*_*L*_ differ, the optimal arrival time of each customer is that defined either by *S*_*E*_ or by *S*_*L*_. Enumerate each of the possible intermediate schedules and evaluate their costs to find the optimum.

Although this algorithm is NP-hard due to the enumeration sometimes required by step (3), *S*_*E*_ and *S*_*L*_ often coincide in practice and seldom differ by more than a few customer arrivals. Computation time is roughly linearly dependent on *N* and *K* in the coincidental case, but the algorithm always requires the evaluation of numerous candidate schedules. Computational efficiency is therefore dependent on a cogent and efficient method for computing the expected cost of a specified appointment schedule, which is the contribution offered by this paper.

## 2. Service Time Model

Suppose that the customer service times are independent, identically distributed random variables with known mean *θ* and variance *σ* ^2^, so the coefficient of variation is *ξ* = *σ*/*θ*. For *ξ* ≤ 1, the service time distribution can be modeled as a mixture of Erlang(*μ*, *r* − 1) and Erlang(*μ*, *r*) distributions with density function

(1) $$\begin{array}{c} f\left(t\right)=\alpha \frac{\mu^{r-1}t^{r-2}}{\left(r-2\right)!}e^{-\mu t}+\left(1-\alpha \right)\frac{\mu^{r}t^{r-1}}{\left(r-1\right)!}e^{-\mu t},\;t\geq 0, \end{array}$$

where 0 ≤ *α* ≤ 1. When the parameters *r*, *α*, and *μ* are chosen such that

(2) $$\begin{array}{c} r=\left\lceil \frac{1}{\xi^{2}}\right\rceil ,\\ \alpha =\frac{\xi^{2}-{\left\{r\left(1+\xi^{2}\right)-r^{2}\xi^{2}\right\}}^{1/2}}{1+\xi^{2}},\\ \mu =\frac{r-\alpha }{\theta }, \end{array}$$

the distribution will have the required mean and variance (see Tijms [19, page 358]). This model is desirable, since *f*(*t*) is always unimodal and is similar in shape to the commonly occurring gamma density function.

For the less typical situation where *ξ* > 1, we can resort to modeling the service time distribution as a mixture of Erlang(*μ*, 1) and Erlang(*μ*, *r*) distributions with density function

(3) $$\begin{array}{c} f\left(t\right)=\alpha \mu e^{-\mu t}+\left(1-\alpha \right)\frac{\mu^{r}t^{r-1}}{\left(r-1\right)!}e^{-\mu t},\;t\geq 0, \end{array}$$

where 0 ≤ *α* ≤ 1. In this case, the required mean and variance can be realized when

(4) $$\begin{array}{c} r=\max\left\{2,\min\left\lceil k:\frac{k^{2}+4}{4k}\geq \xi^{2}\right\rceil \right\},\\ \alpha =\frac{2r\xi^{2}+r-2-{\left(r^{2}+4-4r\xi^{2}\right)}^{1/2}}{2\left(r-1\right)\left(1+\xi^{2}\right)},\\ \mu =\frac{\alpha +r\left(1-\alpha \right)}{\theta }. \end{array}$$

This approach may be reasonable and useful, but should be applied with caution if distribution characteristics beyond the mean and variance are known. Simulation studies have demonstrated that appointment schedule performance is generally insensitive to higher order moments of the service time distribution [20], but these studies are limited to cases where *ξ* ≤ 1.

## 3. Cost Computation

With the chosen service time model, we can compute the expected total cost of a schedule by exploiting the memoryless property of the exponential distribution. The service times are conceptually comprised of exponentially distributed service phases, each with mean duration 1/*μ*. Hence, the state of the system at any time can be completely described by the number of unfinished phases remaining in the system. Let *t*_*k*_ = (*k* − 1)Δ, *t*_*k*_^−^ = lim⁡_*δ*→0_(*t*_*k*_ − *δ*), and *t*_*k*_^+^ = lim⁡_*δ*→0_(*t*_*k*_ + *δ*). Since each phase completion is an event within a Poisson processs, the probability that *s* phases of service remain at *t*_*k*_^−^ given *v* remains at *t*_*k*−1_^+^ can be written as

(5) p(s ∣ v)={e−μΔ(μΔ)v−s(v−s)!,0<s≤v,1−∑m=0v−1e−μΔ(μΔ)mm!,s=0,0,otherwise,

for *k* = 2,…, *K* + 1. Now, let *A*_*k*_ = ∑_*j*=1_^*k*^ *a*_*j*_ and let *q*_*k*_(*s*) be the probability that *s* phases remain at *t*_*k*_^−^. For notational convenience, define a binomial operator $b(i,a,\pi )=\left(\begin{array}{c} a\\ i \end{array}\right)\pi^{i}{\left(1-\pi \right)}^{a-i},0\leq i\leq a,0\leq \pi \leq 1$, and let

(6) z={1,ξ≤1,r−1,ξ>1.

By conditioning on the number of scheduled arrivals that enter the system with fewer than *r* service phases (i.e., with *r* − 1 phases when *ξ* ≤ 1 or with a single phase when *ξ* > 1), we have

(7) $$\begin{array}{c} q_{2}\left(s\right)=\overset{a_{1}}{\underset{i=0}{\sum }}b\left(i,a_{1},\alpha \right)p\left(s\;|\;ra_{1}-zi\right),\;s=0,\ldots ,ra_{1}, \end{array}$$

and, by recursion,

(8) qk(s)=∑i=0ak−1b(i,ak−1,α)×∑m=max⁡⁡(0,s−rak−1+zi)rAk−2qk−1(m)p(s ∣ rak−1−zi+m),       k=3,…,K+1,      s=0,…,rAk−1.

Expected total cost is then given by

(9) C=cw(∑k=1Kak(ak−1)θ2+∑k=2K∑s=1rAk−1qk(s)aksμ)+co(∑s=1rAKqK+1(s)sμ).

The first summation in (9) is equivalent to ∑_*k*=1_^*K*^∑_*j*=1_^*a*_*k*_−1^ *a*_*j*_ *θ* and accounts for waiting due to multiple arrivals scheduled in the same time slot.

The method can be easily extended to accommodate probabilistic customer no-shows. Let *γ*_*k*_ be the probability that a customer actually appears for an appointment scheduled in slot *k*. By conditioning on the number of customers showing up, (7) can be rewritten as

(10) q2(s)=∑j=0a1b(j,a1,γ1)∑i=0jb(i,j,α)p(s ∣ rj−zi),s=0,…,ra1,

and (8) can become

(11) qk(s)=∑j=0ak−1b(j,ak−1,γk−1)∑i=0jb(i,j,α)×∑m=max⁡(0,s−rj+zi)rAk−2qk−1(m)p(s ∣ rj−zi+m),‍    k=3,…,K+1,  s=0,…,rAk−1.

Expected total cost is then computed as

(12) C=cw(∑k=1K ∑j=2akb(j,ak,γk)j(j−1)θ2  +∑k=2K ∑j=1akb(j,ak,γk)∑s=1rAk−1qk(s)jsμ)+co(∑s=1rAKqK+1(s)sμ).

The cost computation procedure can be embedded in an appointment optimization algorithm for statistically identical customers. Unlike more sophisticated and generalized approaches [17], the distribution parameters are trivially calculated and cost computation requires no matrix exponentiation. Schedule optimization can thus be easily performed on a personal computer with common software such as a macroenabled spreadsheet. It should be noted that the cost evaluation of an individual candidate schedule may not require complete computation of *C*; summation and supporting calculations can be terminated as soon as the partial cost of the candidate schedule exceeds the cost of the incumbent.

## 4. Implementation and Performance

Now consider a specific appointment scheduling problem where the number of statistically identical customers is *N* = 10, the number of time slots is *K* = 16, the time slot width is Δ = 0.5, the mean service time is *θ* = 0.75, the service time variance is *σ* ^2^ = 0.25, the show probability is *γ*_*k*_ = 0.95, *k* = 1,…, 16, and the cost of server overtime is estimated at ten times the cost of customer waiting (we notionally set *c*_*w*_ = 1 and *c*_*o*_ = 10, since only relative values affect the optimal schedule). Because *ξ* = 0.6667 < 1, the applicable service time model is given by (1) and the associated parameter values are computed as *r* = 3, *α* = 0.5234, and *μ* = 3.3022. Imbedding the cost computation method within the optimization algorithm described above yields an optimal schedule of

(13) $$\begin{array}{c} S=\left(1,1,1,0,1,1,0,1,0,1,1,0,1,0,1,0\right). \end{array}$$

The associated waiting, overtime, and total costs are 4.8603, 4.9541, and 9.8144, respectively. Optimality is achieved after evaluation of 287 candidate schedules, and complete execution requires less than one second of processing time on a personal computer with a 2.26 GHz processor.

Table 1 illustrates the effect of service time variability by parameterizing on *ξ*. The table quantifies the intuitive positive relationship between variability in service time and the expected cost of the optimal schedule. The number of schedules evaluated diminishes as *ξ* increases, although computation times are longer for very high or low values of *ξ* due to the larger number of exponential phases *r* in the associated service time models.

To further exercise the modeling approach, we can enlarge the baseline problem (with *ξ* = 0.6667) to schedule *N* = 50 customers into *K* = 80 time slots. The cost of the optimal schedule is 51.8026, which is obtained after evaluating 34,162 schedules. Execution time for this very large problem is 483 seconds, which equates to about 0.0141 seconds per schedule evaluated.

## Figures and Tables

**Table 1:** Effect of service time variability.

*ξ*	*r*	Optimal schedule	Total cost	Schedules evaluated	Execution time (sec)
0.125	64	(1,1, 0,1, 1,0, 1,0, 1,1, 0,1, 1,0, 1,0)	1.4072	309	15
0.250	16	(1,1, 0,1, 1,0, 1,1, 0,1, 0,1, 1,0, 1,0)	2.7861	304	2
0.500	4	(1,1, 1,0, 1,1, 0,1, 0,1, 1,0, 1,0, 1,0)	6.7935	287	1
1.000	1	(2,0, 1,1, 0,1, 1,0, 1,0, 1,1, 0,1, 0,0)	15.9581	253	1
1.500	9	(2,1, 1,0, 1,1, 0,1, 0,1, 0,1, 0,1, 0,0)	25.2274	217	1
2.000	16	(2,1, 1,1, 1,0, 1,0, 1,0, 1,0, 0,1, 0,0)	32.8035	189	2
